# 

**Published:** 1898-02-12

**Authors:** 


**Notices of Births, Deaths, and Marriages** are inserted in this column at a charge of 2s. 6d. for an announcement not exceeding four lines.

### NOTICE TO CORRESPONDENTS.

All MS., Letters, Books for Review, and other matters intended for the Editor should be addressed The Editor, "The Hospital," The Hospital Building, 28 & 29, Southampton Street, Strand, W.C.

The Editor cannot undertake to return rejected MS., even when accompanied by stamped directed envelope.

**Notice to Advertisers and Subscribers.**

All Advertisements, Orders for Copies of The Hospital, and Business Communications should be addressed to **THE MANAGER, (Not to The Editor),** The Hospital, The Hospital Building, 28 & 29, Southampton Street, Strand, London, W.C.

The Cost of Subscription, post free, is as follows:—Per week, 2½d. per quarter, 2s. 8d.; per half-year, 5s. 3d.; per annum, 10s. 6d. Foreign:—Per annum, 15s. 2d.: payable, in all cases, in advance.

**NOTICE.—Vol. XXII. of "The Hospital" (April, 1897, to September, 1897), in handsome cloth binding, is now on sale at the Office of this Journal, price 7s. 6d. Cases for binding the Numbers contained in this Volume. 1s. 6d. Index to Vol. XXII., 2½d., post free. The Monthly Part for February is now ready. Price 6d., by post 8d.**

### SCRAPS AND GLEANINGS.

THE board of the Salford Royal Hospital have decided to light the hospital throughout with electric light.

THE Governors of the Royal South Hants Infirmary, at Southampton, have decided to carry out the new building scheme in its entirety.

DURING the year just closed 588 patients were admitted to the wards of the Royal Albert Hospital, Devonport, and 2,326 casualties were treated.

THE average stay of the patients in the Longton Cottage Hospital last year was just one day less than in the previous year, being 25¾ against 26¾ days.

THE number of patients treated in the wards of the Children's Hospital in Temple Street, Dublin, in 1896-7 was 511. There were in addition 5,982 out patients.

THE plans for reinstating and rearranging the existing building of the East Suffolk and Ipswich Hospital, and for building an additional wing has been agreed upon.

THE proposal to give the consulting physicians and surgeons of the Cumberland Infirmary the charge of a certain number of beds in the hospital has been negatived.

IN recognition of the assistance rendered by the *Croydon Guardian* to the Croydon General Hospital during Jubilee year, the editor has been made an honorary life governor.

THE in-patients admitted to the Doncaster Infirmary in 1893-7 number practically the same as those admitted in the previous year, while there have been about 150 fewer out-patients.

THE provident members connected with the Tunbridge Wells Provident Dispensary increased last year by 83, raising the total number to 2,496, of whom 1,674 were above the age of 14.

THE Queen has sent a present of 20 pheasants for the use of the patients in the City of London Hospital for Diseases of the Chest, Victoria Park, E., of which Her Majesty is patron.

IT has been decided by Midhurst parish that neither the "pest-house" property nor the rents accruing therefrom shall be devoted to the Midhurst Cottage Hospital scheme as had been proposed.

THE number of patients treated at the Reigate and Redhill Cottage Hospital during the year ended June 30th, 1897 (268), was about the same as in the previous year. The average stay was 29·2 days.

THERE was a decrease of 91 in the out-patients and an increase of 10 in the in-patients treated at the Derby and Derbyshire Hospital for Sick Children last year. The total number treated was 1,961, viz., 232 in and 1,729 out patients.

H.R.H. PRINCESS LOUISE, Marchioness of Lorne, paid a private visit to the Westminster Hospital and expressed herself as much pleased with the improvements effected by the new operating theatre and new out-patient department.

THE income of the North Staffordshire Infirmary at Hartshill, near Stoke-on-Trent, showed last year a decrease of £236, whilst the expenditure increased by about £313. There is a net adverse balance against the institution of £3,221.

THE accounts of the Boston Cottage Hospital show a deficiency for the past year of £21. The number of patients in the hospital was 176, and 72 out-patients were attended. The falling off in receipts is attributed to the small amount received from collections.

THE work of the Glasgow Maternity Hospital during the past year may be summed up as follows: 2,639 cases were dealt with, 107 students admitted, and 75 nurses trained. The income amounted to £2,018 (inclusive of £465 from legacies), and the expenditure to £2,076.

THE medical board of the Salford Royal Hospital in their last report direct attention to the fact that there is a progressively growing need for increased hospital accommodation in Salford, which demands grave consideration, and which, in the near future, will have to be dealt with. The increased number of patients is partly the result of increase of the resident population, but beyond this there is a considerable contingency of serious cases due to the proximity of the hospital to the Ship Canal.

### THE STUDENT OF MEDICINE.

**APPOINTMENTS.**

H. Armstrong, M.B., Ch.B.Vict., appointed House Surgeon to the Infirmary for Children, Liverpool. R. Browne, M.D.-Dub., F.R.C.S.I., appointed Superintendent Medical Officer of Health for Rathmines (Dublin) B. Dyball, M.B., B.S.Lond., F.R.C.S., L.R.C.P.Lond., appointed Resident Casualty Officer at the Leeds General Infirmary. Mr. M. M. Harley, appointed Medical Officer of Health to the Newport Pagnell Urban District Council. G. Hope, L.R.C.P.Lond., M.R.C.S.Eng., D.P.H.Eng, appointed Medical Officer for the Seventh District of the Brentford Union. O. Horrocks, L.M.&S.Madras, L.R.C.P.&S., F.R.C.S.Eng., appointed District Surgeon, Sierra Leone. J. W. H. Jellett, M.D.Dub., M.B., B.Ch., appointed Medical Officer of Health to the Heavitree Urban District Council. G. E. Middlemist, M.B., appointed Second Assistant Medical Officer to the County Asylum, Dorchester. A. G. Miller, M.D., F.R.C.S.Edin., appointed an additional Examiner in Clinical Surgery at the University of Edinburgh. A. E. Normington, M.B., Ch.B Vict., appointed Medical Officer of the Workhouse of the Stoke upon-Trent Union. C. J. Palmer, L.S.A., appointed Medical Officer for the No. 2 District of the Mansfield Union. A. Stanley, M.D., B.S.Lond., D.P.H., appointed Medical Officer of Health for Shanghai. J. F. Strickland, M.B., C.M.Edin., appointed Assistant House Surgeon to the Infirmary for Children, Liverpool. F. Warner, M.D.Lond., appointed an additional Examiner in Materia Medica at the University of Aberdeen. J. P. Watt, M.A., M.B., appointed an additional Examiner in Medical Jurisprudence and Public Health at the University of Aberdeen. H. W. Webber, M.D., M.S.Lond., appointed Assistant Surgeon to the South Devon and East Cornwall Hospital, Plymouth. E. J. Whitaker, M.B., C.M.Edin., appointed Medical Officer of Health to the Blymhill Rural District Council. T. F. Woodhead, M.R.C.S.Eng., L.R.C.P.Lond., appointed Medical Officer for the Meltham District of the Huddersfield Union. H. E. Belcher, L.R.C.P.Lond., M.R.C.S., appointed Medical Officer for the West Bridgford District of the Basford Union. Dr. J. M. Bennett, appointed Medical Officer for the Ruddington District of the Basford Union. J. Buchanan, M.B., R.U.I., M.Ch., appointed Medical Officer for the Watford District of the Watford Union. J. N. Cook, M.R.C.S., L.R.C.P., appointed Medical Officer of Health for Calcutta. T. G. Dickson, L.R.C.P., L.R.C.S.Edin., L.F.P.S.Glasg., appointed Medical Officer for the Radnorshire District of the Hay Union. F. A. Elkins, M.D., appointed Medical Superintendent of the Metropolitan Asylum at Leavesden, Herts. G. R. Green, L.R.C.P.Edin., M.R.C.S.-Eng., appointed Medical Officer of Health to the Ripon City Council. F. Greaves, M.R.C.S., appointed Assistant House Surgeon to the Derbyshire Royal Infirmary. F. A. Hallsworth, L.R.C.P.Lond., M.R.C.S.Eng., appointed Medical Officer for the Bodenham District of the Leominster Union. G. L. Hanwell, M.R.C.S., L.R.C.P., appointed Clinical Assistant to Out patients at Chelsea Hospital for Women. W. Harding, M.D.Edin., M.R.C.P., appointed Medical Superintendent, Northampton County Asylum, Berry Wood. H. Horrocks, M.D.Lond., D.P.H., appointed Assistant Pathologist and Bacteriologist to the Public Hospital, Perth, Australia. G. Hudson, M.B., C.M Edin., appointed Medical Officer for the Workhouse and the Worlington District of the Mildenhall Union. W. Lockwood, M.D.Edin., appointed Honorary Surgeon to the Royal Halifax Infirmary. J. R. Lord, M.B., C.M.Edin., Resident Clinical Assistant at the Joint Counties Asylum, Carmarthen, South Wales, appointed an Assistant Medical Officer at the London County Asylum, Hanwell. P. R. Lowe, B.A., B.C.Camb., appointed House Surgeon to the Derbyshire Royal Infirmary. Dr. J. G. Milnes, appointed Medical Officer for the Gislingham District of the Hartismere Union. A. E. Morison, M.B., C.M.Edin., F.R.C.S.Edin., appointed Honorary Surgeon to the Hartlepool Hospital. W. H. I. Sellers, M.B., C.M.Edin., M.R.C.S.Eng, appointed Honorary Surgeon to the Preston and County of Lancaster Royal Infirmary. J. Shepheard, B.A.Camb., M.R.C.S., L.R.C.P., appointed Medical Officer of Health to the North Walsham Urban District Council. Dr. E. Smith, appointed Medical Officer of the Brownlowhill Workhouse of the Parish of Liverpool.

